# Micro Non-Uniform Linear Array (MNULA) for Ultrasound Plane Wave Imaging

**DOI:** 10.3390/s21020640

**Published:** 2021-01-18

**Authors:** Yujia Tang, Zhangjian Li, Yaoyao Cui, Chen Yang, Jiabing Lv, Yang Jiao

**Affiliations:** 1 Division of Life Sciences and Medicine, School of Biomedical Engineering (Suzhou), University of Science and Technology of China, Hefei 230026, China; tyj95@mail.ustc.edu.cn (Y.T.); mochen93@mail.ustc.edu.cn (C.Y.); 2 Suzhou Institution of Biomedical Engineering and Technology, Chinese Academy of Sciences, Suzhou 215163, China; lzjian@mail.ustc.edu.cn (Z.L.); cuiyy@sibet.ac.cn (Y.C.); lvjb@sibet.ac.cn (J.L.)

**Keywords:** non-uniform linear array, plane wave imaging, near field, sound field, imaging quality, endoscope

## Abstract

Ultrasound plane wave imaging technology has been applied to more clinical situations than ever before because of its rapid imaging speed and stable imaging quality. Most transducers used in plane wave imaging are linear arrays, but their structures limit the application of plane wave imaging technology in some special clinical situations, especially in the endoscopic environment. In the endoscopic environment, the size of the linear array transducer is strictly miniaturized, and the imaging range is also limited to the near field. Meanwhile, the near field of a micro linear array has serious mutual interferences between elements, which is against the imaging quality of near field. Therefore, we propose a new structure of a micro ultrasound linear array for plane wave imaging. In this paper, a theoretical comparison is given through sound field and imaging simulations. On the basis of primary work and laboratory technology, micro uniform and non-uniform linear arrays were made and experimented with the phantom setting. We selected appropriate evaluation parameters to verify the imaging results. Finally, we concluded that the micro non-uniform linear array eliminated the artifacts better than the micro uniform linear array without the additional use of signal processing methods, especially for target points in the near-field. We believe this study provides a possible solution for plane wave imaging in cramped environments like endoscopy.

## 1. Introduction

Ultrafast ultrasound imaging has greatly influenced the way ultrasound imaging has developed, since Fink and co-authors put forward coherent plane wave compounding (CPWC) in 2009, which made ultrafast ultrasound imaging reality [1,2,3]. In CPWC, a picture is produced by controlling all the elements of a linear array to transmit sound waves and receive reflected data at the same time [4]. In an ideal situation, the transmitted wave front is a flat plane. It has to be mentioned that the actual near-field transmitted by a uniform linear array (ULA) is distinguished from the ideal plane wave premised in CPWC [5]. To make the imaging quality equivalent to the ideal situation, the actual transmitted wave should remain flat during the propagation process. After receiving data from the linear array, the beamforming process can be described as selecting data to corresponding actual positions. In this way, plane wave imaging creates images with a high speed of up to 5 Kf/s [4].

Ultrasound plane wave imaging technology has been applied in different clinics due to its advantages of fast imaging speed and high imaging quality. This technology was first utilized to image the transient propagation of shear mechanical waves in real time [4,6]. Later in 2011, ultrasound plane wave imaging technology was put into use for blood flow Doppler imaging [7,8]. Doppler imaging and transient elastography can provide more diagnostic information when applied in an endoscopy [9,10,11]. All these applications are based on the good imaging quality of plane wave imaging in an endoscopy. However, all these applications are rarely studied in the endoscopic environment. On the one hand, in existing applications, the near-field contains the surface of skin, from which imaging targets still need distance, but a miniaturized linear array is strictly required in the endoscopic environment [12]. The kind of linear array used in an in vitro environment usually has the following aspects: its structural distribution follows the original ultrasonic linear array design laws, and the parameters of each array element are equal. We call this a ULA [13]. Because of the need for adequate space for a linear array transducer in an in vitro environment, the number of elements of a ULA need to get a wider field of view is usually 128 or more [14]. Meanwhile, in the endoscopic environment, the number of elements has to be reduced to meet the small size requirements.

In order to solve these issues in the endoscopic imaging environment, a solution needs to be proposed. Here, we designed a new linear array structure for ultrasound plane wave imaging that was inspired by the antenna linear array. In the area of antenna linear arrays, existing work has shown that there is a special type of linear array that can achieve a better signal resolution and minimize the sidelobe level of beam patterns [15,16,17]. Additionally, it provides practical advantages such as reductions in size [18,19]. An ultrasound transducer is a kind of antenna array that transmits and receives signals [20]. This is how we got the new idea of re-designing the structure of an ultrasound linear array transducer. The small-sized ULA used in the endoscopic environment is called a micro uniform linear array (MULA). Thus, we named the special kind of linear array a micro non-uniform linear array (MNULA). In previous work [21], we concluded that the new structure brought a lot of changes to the existing kind of linear array transducer, including the distribution of each element and the combination of different parameters of different elements. For this paper, we tried to verify whether the new structure design could improve the transmitted plane wave sound field and its imaging results produced by CPWC through simulations and experiments.

The rest of the paper is organized as follows: in Section 2, we demonstrate the sound field calculation of linear array transducers and models of the MNULA. CPWC for the MNULA is also introduced in this part. In Section 3, we simulate the sound field and imaging process to verify the effect of the MNULA. The actual imaging quality is studied in Section 4. At last, we conclude the results from the completed research work and discuss its possible applications in the future.

## 2. Methods

### 2.1. Transducer Model of MNULA

A ULA is mostly chosen at present to transmit plane waves. However, the regular arrangement of elements makes adjacent elements have the same phase, which leads to high sidelobes. When we eliminate the uniformity of a transducer, it can be predicted that the sidelobe will be suppressed. Meanwhile, for an ultrasonic linear array transducer, there is little information about the re-design of the structure. Almost no mature theory about the MNULA exists. A symmetrical distribution whose interelement spacings present an increasing trend from the middle to both sides is common in antennas. The element spacings usually has the following limits [18]:

(1) $$0.5\lambda \leq d_{p}-d_{p-1}<0.5\lambda +\Delta$$

where $d_{p}$ is the spacing between the p-th element and the (p−1)-th element, λ is the wavelength of the transmitted wave, and Δ is the space broadening factor.

**Figure 1:**
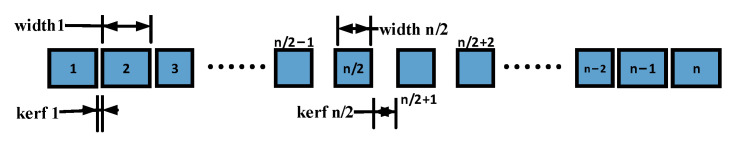
An example of a linear array.

Therefore, we applied the distribution to the design process of the MNULA, and we made some adjustments according to the structural parameters of the linear ultrasound array. The parameters were divided into two types—the width of the array element and the spacing between adjacent array elements, the last of which we can also call kerf. One example of linear arrays with *n*/2 types of structural parameters is demonstrated in Figure 1.

**Figure 2:**
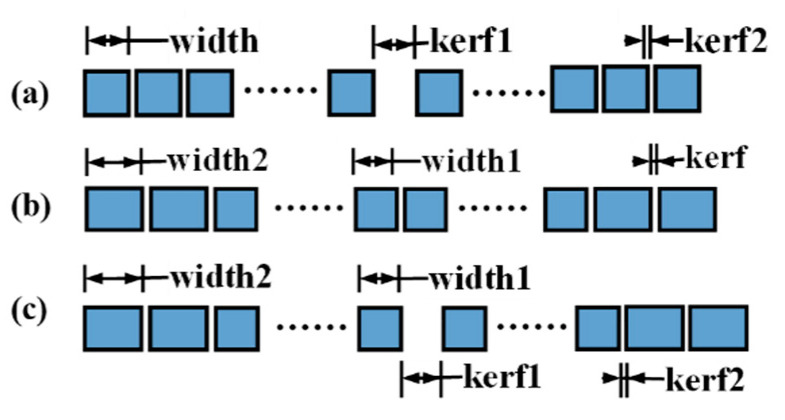
Three types of micro non-uniform linear arrays (MNULAs). (**a**) An MNULA with different kerfs (spacings between adjacent array elements), (**b**) an MNULA with different widths, and (**c**) an MNULA with different kerfs and widths.

While numerical values and the distribution of variables remain to be further studied, we can easily designate three types of arrays from these acknowledged structural parameters. Here, three combinations of the parameters shown in Figure 2 were chosen to verify the tendency of the sound field of the MNULA [8].

In the simulation part, we chose the three theoretical transducer models for verification, and their parameters were adjusted based on the existing laboratory-made micro uniform linear array transducer.

### 2.2. Sound Field of Linear Array

The sound field calculation has an important place in an analysis of the shape of a wave-front. Far-field directivity provides the two-dimensional information of a sound field, which means the distribution of the isobaric surface in a certain plane. The far-field directivity provides a guideline for the design of a linear array.

**Figure 3:**
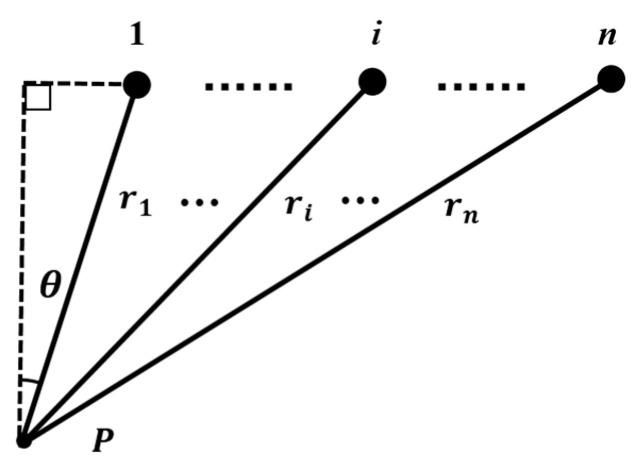
The point P(r,θ) in the sound field and its relative position to n elements of the linear array.

An n-element linear array consists of *n* rectangular transducers with widths of $a_{1},a_{2},a_{3},\cdots ,a_{n}$ and a length of *L*. These elements are arranged with kerfs of $d_{1},d_{2},d_{3},\cdots ,d_{n-1}$. The field point P(r,θ) and its relative position to the elements are described in Figure 3.

In order to get the far-field directivity of a linear array, the sound pressure of a single rectangular planar transducer has to be calculated. Take the first element of the linear array in Figure 3 for an example. The sound pressure of point P(r,θ) is expressed in Equation (2).

(2) $$p(r,\theta ,t)={\left(\frac{p_{0}}{r}\right)}^{\frac{1}{2}}\frac{\sin(\frac{ka\sin\theta }{2})}{\frac{k\sin\theta }{2}}\cdot e^{\frac{-jka\sin\theta }{2}}\cdot e^{j(\omega t-kr)}$$

where $p_{0}$ is the initial pressure, r is the distance between the field point and transducer element, ω is the angular frequency, and t is a certain time at the process of propagation. The variable ‘*k*’ is expressed as

(3) $$k=\frac{2\pi }{\lambda }=\frac{2\pi f}{c}$$

where λ is the wavelength, f is the center frequency of the array, and c is the sound speed.

When all the elements receive the same excitation and then produce the same response, the total sound pressure of the linear array is the sum of the sound pressures of all the array elements. It can be expressed in Equation (4), which is achieved from adding the sound pressure from the first to the *n*th element.

(4) $$\begin{array}{ll} P(r,\theta ,t) & =\sum_{i=1}^{n}p_{i}(r_{i},\theta_{i},t)\\ & \;={\sum_{i=1}^{n}\left(\frac{p_{i}}{r_{i}}\right)}^{\frac{1}{2}}\cdot \frac{\sin(\frac{ka_{i}\sin\theta }{2})}{\frac{k\sin\theta }{2}}\cdot e^{\frac{-jka_{i}\sin\theta }{2}}\cdot e^{j(\omega t-kr_{i})} \end{array}$$

where $p_{i}$ is the sound pressure produced by the *i*-th element and $r_{i}$ is the distance between the field point and the *i*-th transducer element. The value of $r_{i}$ (1<i≤n) can be calculated by Equation (5):

(5) $$r_{i}=\sqrt{r_{1}^{2}+{\left[\sum_{j=1}^{i-1}\left(a_{j+1}+d_{j}\right)\right]}^{2}+2r_{1}\cdot [\sum_{j=1}^{i-1}\left(a_{j+1}+d_{j}\right)]\sin\theta }$$

To emphasize the distribution of sound field, the directivity function neglects the influence of amplitude and can be expressed in Equation (6). $\theta_{max}$ is the direction where the pressure amplitude reaches the maximum. In the sound field of a plane wave, $\theta_{max}$ is 90°.

(6) $$H(\theta )=\left|\frac{p(r,\theta ,t)}{p(r,\theta_{\max},t)}\right|$$

It should be acknowledged that the sound field of an MNULA still satisfies the fundamental superposition theorem mentioned earlier. The difference between an MNULA and an MULA is the uniformity of their structural parameters.

### 2.3. Coherent Plane Wave Compounding Imaging for MNULA

The original CPWC algorithm is suitable for an MULA. However, when it comes to completing the whole process with an MNULA, the non-uniform distribution of structural parameters have to be taken into consideration. In this way, we propose a new beamforming method for an MNULA based on coherent plane wave compounding imaging.

**Figure 4:**
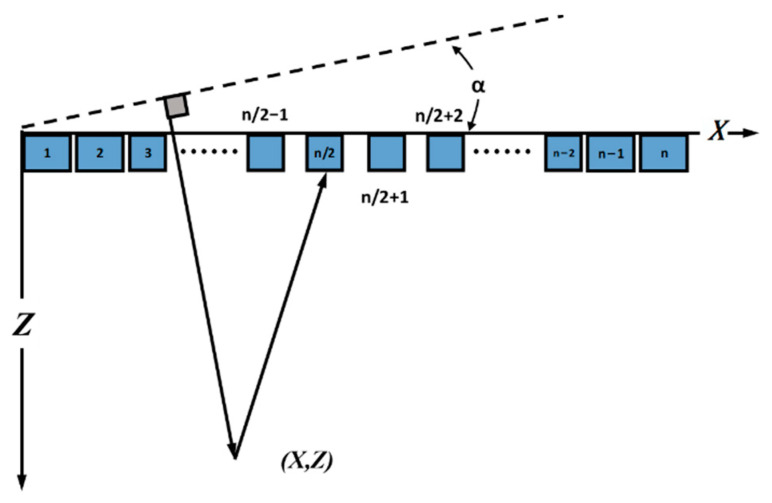
The geometric coherent plane wave compounding (CPWC) relationship that the MNULA satisfies. The plane wave is with an angle α.

On the basis of structural parameters in part B, the positions of *n* elements can be expressed as $x_{1},x_{2},\ldots \ldots ,x_{n}$. The time delay for each element to transmit a plane wave with an angle α is calculated with the non-uniform distribution $x_{1},x_{2},\ldots \ldots ,x_{n}$ as Equation (7). To better explain the geometric relationship between the scatter point (*x*, *z*) and wave front, Figure 4 shows the geometric relationship.

(7) $$[\begin{array}{ccccc} \tau_{1} & \tau_{2} & \cdots & \tau_{n-1} & \tau_{n} \end{array}]=\frac{[\begin{array}{ccccc} x_{1} & x_{2} & \cdots & x_{n-1} & x_{n} \end{array}]\cdot \sin\alpha }{c}$$

From the geometric figure, the time for the wave front to travel from starting line to point (*x*, *z*) can be figured out with Equation (8).

(8) τ(α,x,z)=(zcosα+xsinα)/c

To travel back to the element n in the position $x_{n}$, the plane wave needs time expressed in Equation (9):

(9) $$\tau (x,z,x_{n})=\sqrt{z^{2}+{\left(x-\sum_{i=1}^{n}x_{i}\right)}^{2}}/c$$

The image of CPWC for an MNULA is obtained by processing the data with the principles mentioned above. The readers are referred to [4,6] for further descriptions of the CPWC for a ULA.

## 3. Simulation

### 3.1. Sound Field

#### 3.1.1. Settings

We used the Acoustics, Solid Mechanics, and Electrostatics modules of COMSOL Multiphysics 5.4 to do the simulations. The simulation model selected the method of far-field directivity to figure out the distribution of sound field. The model utilized the piezoelectric effect to produce a sound field. The pressure distribution was calculated during the propagation process in a given region. The material of the region was selected as water, where the speed of the ultrasound wave was rather close to that in human body.

**Figure 5:**
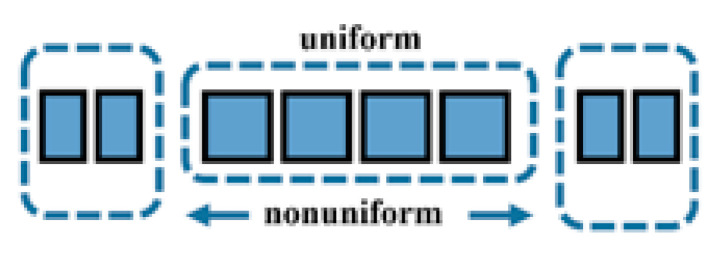
Regional distribution of an MNULA.

The model of an eight-element linear array with a center frequency of 12 MHz was chosen to be verified. On the one hand, a limited number of elements could form the basic shape of the sound field. On the other hand, the number could also reduce the possibility of errors caused by an excessive number of elements during the iterative calculation. After considering several kinds of distributions of elements, the regional settings shown in Figure 5 were adopted for their symmetrical structure and low complexity.

The structural parameters of the middle part were the same as the MULA. The elements in the non-uniform region satisfied the non-uniform parameters, which can be found in Table 1. The parameters for the MULA were based on the existing laboratory production.

Following the above-described settings, the structural parameters of the ultrasonic linear array are summarized in Table 1. In the table, kerf1 refers to the spacing between the adjacent elements in the uniform part and kerf2 refers to the spacing between the remaining elements on both sides. Width1 and width2 refer to the structural parameters of the same areas as the kerfs.

In order to obtain the sound pressure distribution of the field within a small distance of the transducer surface, a high accuracy of calculation was required. Therefore, we divided the propagation medium into meshes with smaller unit sizes. The largest unit size was set at up to 20 μm, and the minimum unit size was 1.65 μm. Meanwhile, the internal mesh of the piezoelectric transducer could be increased to some extent, so the size was set to 60 μm to simplify the calculation inside the transducer. This way of allocating the sizes of the mesh saved computing resources and reduced the probability of error in iterative calculations.

#### 3.1.2. Results

**Figure 6:**
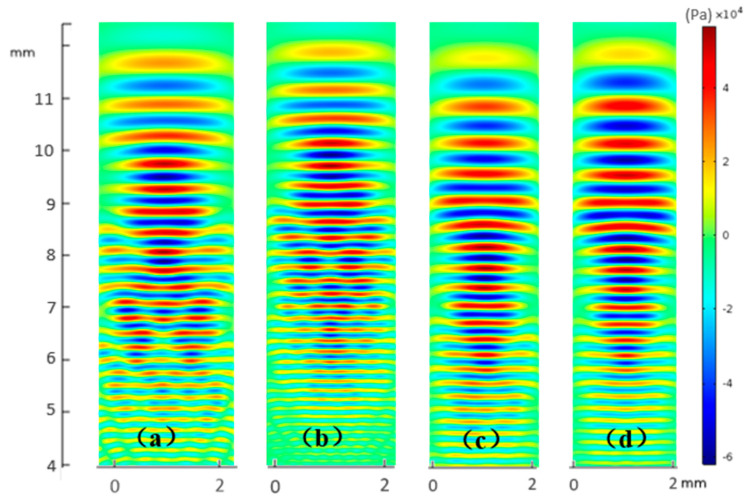
Sound field of transducers with different non-uniformities at the same time. (**a**) A uniform linear array transducer, (**b**) a linear array with various kerfs, (**c**) a linear array with various widths, and (**d**) a linear array with various widths and kerfs.

The results are shown as Figure 6. After a large number of simulations, some preliminary features could be described. When the width and kerf of some elements were changed at the same time, the sound field was greatly improved in both the near and far fields. Regarding the geometric distribution of sound waves transmitted by a single transducer, the farther away from the surface of transducer, the closer the shape of sound waves was to the ideal plane wave. However, the area close to the surface of the transducer was discontinuous in the distribution of isobaric surface and could not form a plane wave. It could be observed that the near field was rather closed to the sound sources located on the surface of the array. The phase differences from different sound resources caused mutual interference in the wave front, thus resulting in a large difference in horizontal distribution. This is why the near sound field of the MULA seemed discontinuous. For the MNULAs in Figure 6b–d, the mutual interference in the wave front was weakened when the widths and kerfs were changed in the MNULA. The MNULA in Figure 6d, which had various widths and kerfs, had the best planarity in the near field for its weak mutual interference. From the results obtained by different types of MNULAs, it could be seen that the shape of the sound field was smoother and the energy level remained at the same magnitude as the number of non-uniform parameters increased.

### 3.2. Imaging

#### 3.2.1. Settings

From the theoretical analysis of CPWC, it was acknowledged that the flatter the wave front in the near field was, the more accurate of plane wave imaging algorithm became. A wider field and more concentrated energy could also bring improvements. Accordingly, the significant improvement of the image quality of the MNULA should be reflected in the near field. As such, the imaging was verified and the quality was validated with the Field II simulation software.

The structural parameters of four different types of linear arrays satisfied the conditions shown in Table 1. The center frequency remained at 12 MHz. We expanded the number of elements to 32, and the proportion remained the same. This made the field of view wider. We adopted seven angles of plane waves ranging from −5° to 5° to achieve the majority of ideal plane wave situations [8].

**Figure 7:**
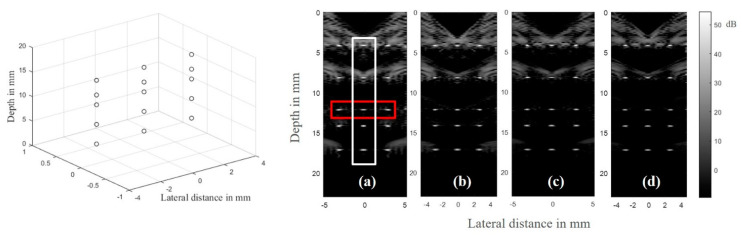
The phantom and comparisons between simulation results of uniform and non-uniform linear arrays at different depths arranged in the order that corresponds to the sound simulation results: (**a**) The result of different depths of the MULA, (**b**) the MNULA with various widths, (**c**) the MNULA with various kerfs, and (**d**) the MNULA with various kerfs and widths.

When it came to the phantom settings, phantoms containing points and cysts were selected. In order to observe the image at different positions, scatter points were set at different depths and widths of the phantom. The point phantom had several scatters with a symmetric structure, as shown in Figure 7. The structure could provide a better comparison of the results of the MULA and the MNULA. To make the simulation phantom closer to a real environment, the simulated cyst phantom was selected for its large amount of scatters and complex shapes. In this situation, the cyst phantom could better simulate the imaging quality of the MNULA.

#### 3.2.2. Point Phantom

The simulation results are shown as Figure 7. Apparent changes occurred in two areas: 1~10 and 12~18 mm. The imaging results are arranged according to the order of transducers in table. The columns are, from left to right, MULA no.1, no.2, and no.3.

When it came to the depth dimension in one image, a number of symmetrical artifacts appeared on both sides of target points, and their distribution area was obviously larger than the second region. The first region was rather close to the surface of the transducer, and its sound pressure on axis showed strong fluctuations between maximum and minimum values. All these made the area in 0~10 mm not an ideal plane wave imaging environment. In the second area, imaging quality was significantly improved because of the stabilization of the sound field.

Compared to the imaging results of the MULA, the different types of MNULAs differed in terms of improvement. All the MNULAs suppressed the artifacts on both sides of points in different depth areas. Among them, the last linear array with the no.3 non-uniform parameter combination had the best performance. For areas in 0~10 mm, we could clearly distinguish target points from artifacts. To the naked eye, the middle of the three columns had the most obvious level of artifact elimination. Even the brightness in the area of 12~18 mm was slightly decreased and the artifacts on both sides were radically eliminated, which was equivalent to the quality obtained by some complex filtering algorithms [22,23,24].

**Figure 8:**
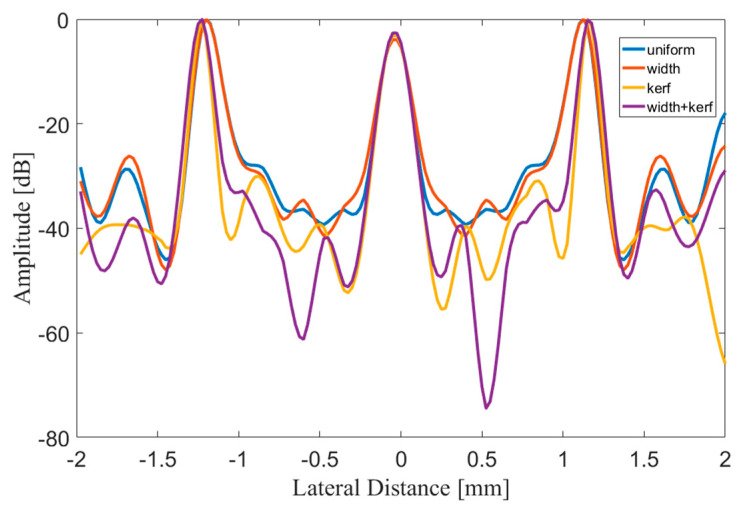
Lateral envelope of echo signals of the points inside the red rectangle from the uniform linear array (blue line), the MNULA with different widths (orange line), the MNULA with different kerfs (yellow line), and the MNULA with different kerfs and width (purple line).

**Figure 9:**
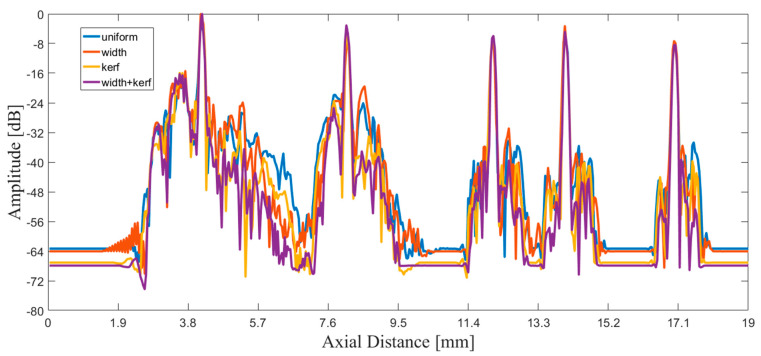
Axial envelope of echo signals of the point inside the white rectangle from uniform linear array (blue line), the MNULA with different widths (orange line), the MNULA with different kerfs (yellow line), and the MNULA with different kerfs and width (purple line).

The points inside the red rectangle and the white rectangle were calculated for lateral and axial envelopes in Figure 8 and Figure 9, respectively. It could be observed that the MNULA with various widths and kerfs had the lowest level for artifacts around the target points, which was consistent with the imaging comparison shown in Figure 7.

#### 3.2.3. Cyst Phantom

**Figure 10:**
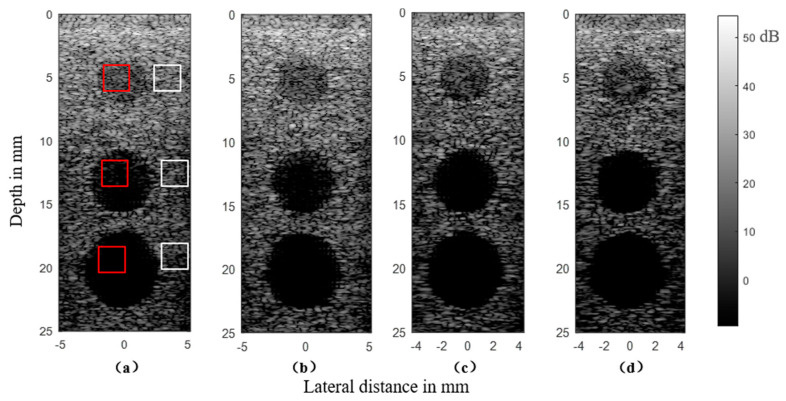
Simulation results of a cyst phantom from: (**a**) an MULA, (**b**) an MNULA with various kerfs, (**c**) an MNULA with various widths, and (**d**) an MNULA with various kerfs and widths.

The simulated cyst phantom consisted of three circle cysts lying in the tissue. The 4 mm-diameter cyst was at the depth of 5 mm, the 6 mm-diameter cyst was located at the depth of 13 mm, and the 7 mm-diameter cyst was at the depth of 21 mm. The simulation results of the cyst phantom are shown in Figure 8. For the micro linear array, the majority of artifacts appeared in the near field. In Figure 10a it can be seen that the artifacts distributed in the near field of the MULA affected the observation of the phantom shape. The cysts located at the depths of 5 and 13 mm were much more affected than that located at 20 mm because the cyst at the depth of over 20 mm was in the far field and the MULA could get a clear outline of the it.

**Figure 11:**
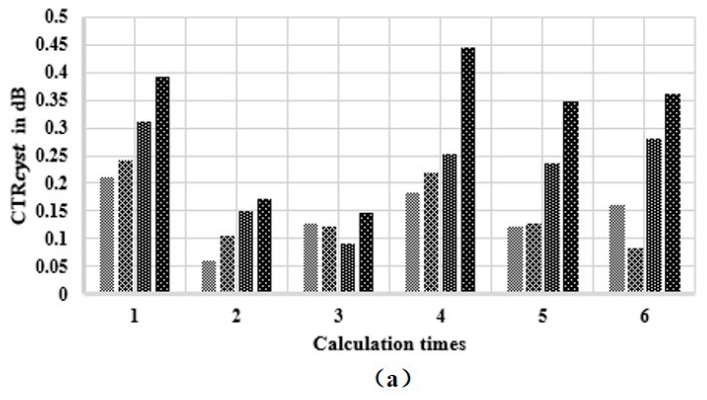

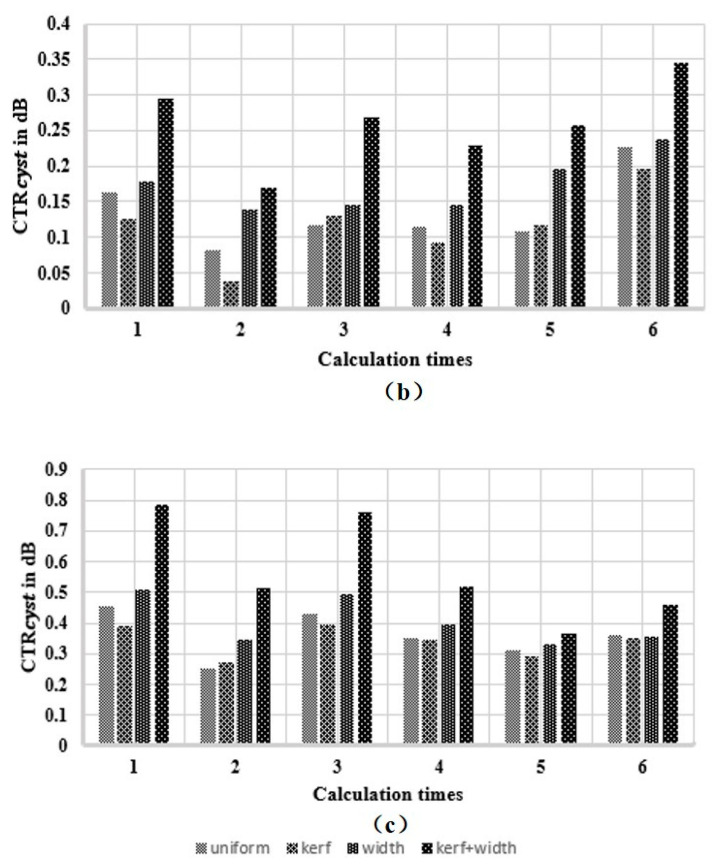
The contrast rations of the simulation results: (**a**) The cyst located at the depth of 5 mm, (**b**) the cyst located at the depth of 13 mm, and (**c**) the cyst located at the depth of 20 mm.

From Figure 10a–d, it can be observed that the clarity of the cysts got a gradual improvement for different types of MNULAs. To evaluate the improvement, we selected a contrast to describe the difficulty of distinguish cysts from the background. The contrast was described as the ratio expressed as the following equation:

(10) $${CTR}_{cyst}=\;-10log_{10}\frac{\mu \left(RF_{inside}\right)}{\mu \left(RF_{outside}\right)}/log_{10}\left(\sigma^{2}\left(RF_{outside}\right)\right)$$

where μ means the mean value and$\;\sigma^{2}$ means the variance value. The higher the contrast was, the less artifacts lay inside the cyst. The red square indicates the region of interest (ROI) inside the cyst, and the white square indicates the background area at the same depth. Six rectangular regions with a length of 15 pixels and a width of 15 pixels were selected randomly in the cyst and background, respectively. The operation was repeated on all the three cysts for the consideration of depth. The results are shown in Figure 11.

The figure shows that different types of MNULAs eliminated artifacts to varying extents. The MNULA with different kerfs eliminated artifacts slightly better than the MULA. When it came to the MNULA with different widths, its contrast was obviously higher than the MULA’s, as seen in Figure 11b,c, thus leading to the artifacts caused by the linear array being gradually eliminated, as seen in Figure 10c. The MNULA with different kerfs and widths had the highest contrast, which indicated that this kind of MNULA had the least artifacts inside the cysts at different depths. In the imaging results obtained by the MNULA with both non-uniform distributed kerfs and widths, the artifacts inside the cyst basically disappeared, which meant this type of MNULA had the strongest elimination of artifacts and had the best imaging quality in the near field. This phenomenon was also consistent with the results of the target phantom and sound field simulation.

The figure also shows that the speckle was widened with the improvement of the non-uniformity of the MNULA. When the structural parameters with the non-uniform distribution of the MNULA increased, the speckle was widened, especially in the far field. We think that this may have been caused by the decrease of the sensitivity for the MNULA in the far field. In the sound field simulation, a comparison of the results showed that the sound pressure of the MNULA at the distance of 10 mm was less than that of the MULA. The speckle could not be avoided when the plane wave ultrasound imaging was applied to the MNULA, but this was not serious and could be improved with some beamforming methods for plane wave ultrasound imaging.

Thus far, all the simulation parts showed that the non-uniform linear array achieved a good effect on the near field artifacts during coherent compound plane wave imaging.

## 4. Experiment

### 4.1. Transducer Quality Evaluation

**Figure 12:**
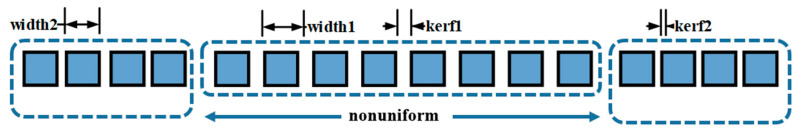
The schematic of the home-made MNULA.

The existing technology of the laboratory needed to be considered before we determined the parameters of the linear array. The number of elements was chosen as 16 in order to be compatible with the subsequent laboratory driver hardware. Considering the existing manufacturing technology in the laboratory, the following parameters were selected for experimental verification in the experimental design. The structural parameters are shown in Figure 12, and they satisfied the numbers in Table 2.

**Figure 13:**
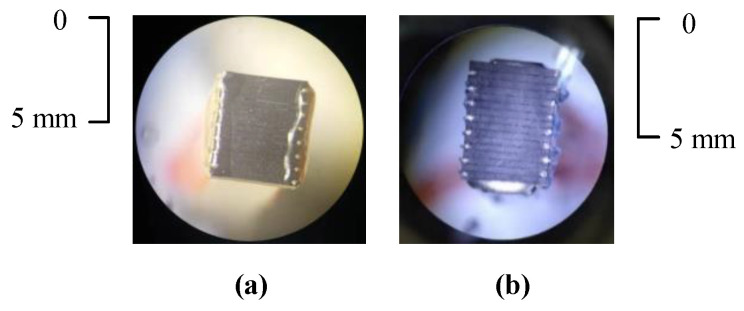
The linear array transducers made by a lab. (**a**) An MNULA and (**b**) an MULA.

Before the transducers were coated with a protective layer, the overall size was as small as 4×5 mm, and the kerfs on the surface of the graphite were clearly visible under a microscope. Their surfaces are shown in Figure 13.

In order to ensure that only the structural parameters of the transducer affected the final imaging results, the performance of the elements of the uniform MULAs and MNULAs was tested and carefully compared to make sure that manufacturing process was kept consistent when making different MULAs. In this way, the quality of the transducers was kept consistent.

**Figure 14:**
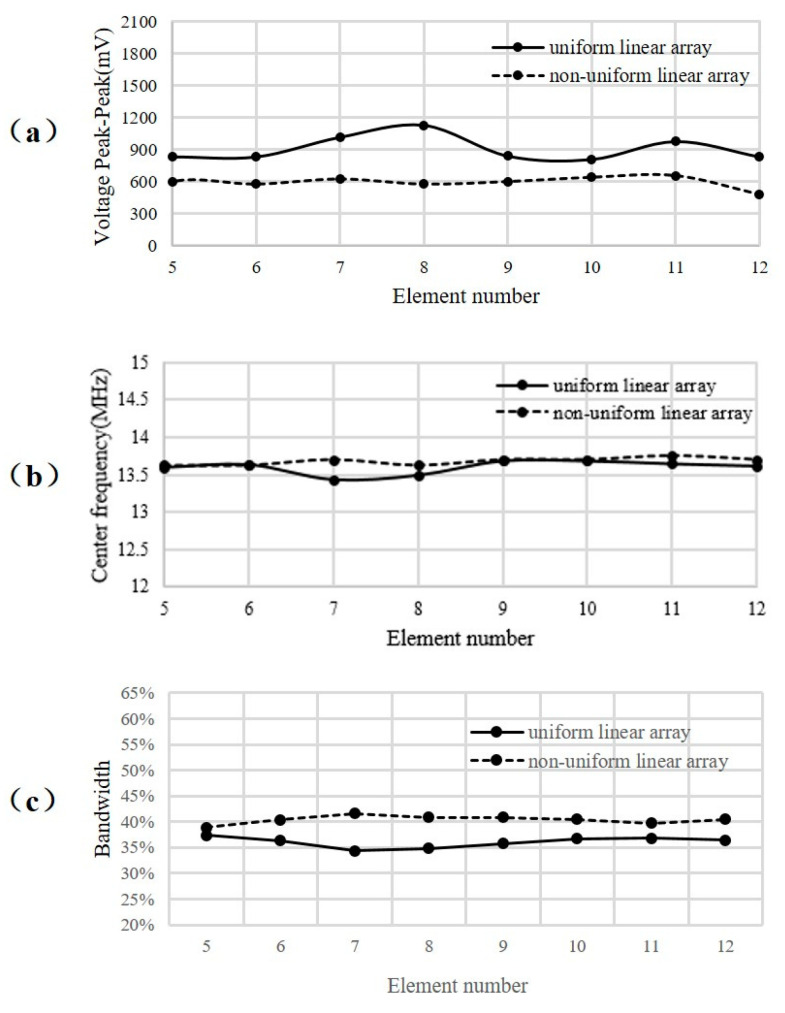
Comparisons of peak-to-peak voltage and center frequency between the MULA and the MNULA. (**a**) Peak-to-peak voltage, (**b**) center frequency, and (**c**) bandwidth at −6 dB.

The amplitude and frequency distribution of the elements in the uniform part are shown in Figure 14. For peak-to-peak voltage, the difference between the MULA and the MNULA was around 400 mV. The center frequency values remained at an average of 13.5 MHz. For bandwidth, the MULA remain had an average of 35% and the MNULA had an average of 40%. We think this was a tolerable difference between the different arrays. For the MULA and the MNULA, different elements kept a strong consistency in peak-to-peak voltage, center frequency, and bandwidth. It can be concluded that an MULA and an MNULA with the same manufacturing processes have similar qualities. In this way, imaging conditions for different transducers are promised to be the same.

### 4.2. Experimental Setup

The experiment platform was built on a Vantage 64 LE HF (Verasonics, Kirkland, America). In the control manuscript, the same voltage amplitude was used to simultaneously excite all the elements in which plane wave was transmitted. A laboratory-made phantom was designed to satisfy the requirement of point target, which had three fish lines stretched tight in the water. The 0.2 mm-diameter fish lines had an axial spacing of 3 mm and a lateral spacing of 1.5 mm. A schematic diagram is shown in Figure 13a. We imaged their transverse sections as three target points of *a*, *b*, and *c*.

In the imaging experiment, a high-precision fixture was utilized to fine-tune the imaging angle of the transducer. After the ideal angle was obtained, it was fixed to ensure that the error caused by the position of different transducers and the target point was minimized so the imaging quality would not be effected.

### 4.3. Results and Analysis

**Figure 15:**
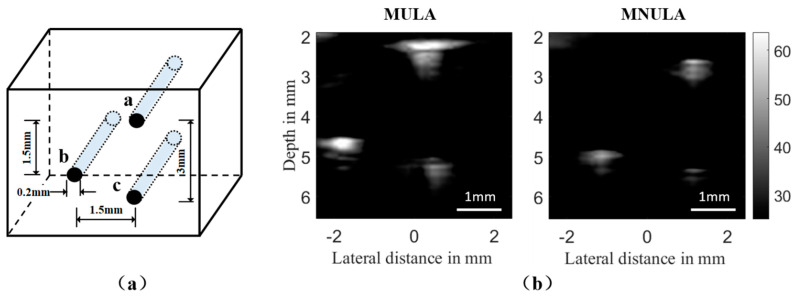
The laboratory-made phantom and comparisons between the experimental results of the MULA and the MNULA. (**a**) Enlarged schematic diagram for the phantom; (**b**) the enlarged imaging results of the phantom.

We directly chose the imaging results from the Vantage user interface. Apart from structural parameters, all the beamforming and imaging setups kept the default Vantage 64 LE HF system settings. The default mode for the bandwidth of the receive data was 200%, and all of the 4f_c (center frequency) samples were kept in the final output. The same time gain compensate (TGC) curve was applied to the imaging experiment for the MULA and the MNULA. Still, changing the arrays brought about a 0.3 mm offset. In this way, the imaging improvements brought by structure modification could be compared under the same beamforming and imaging processes. Due to the narrow field caused by the small size of transducers, the target points were enlarged and compared, as shown in Figure 15.

In the B-mode image from the Vantage 64 LE HF, the fish lines located at the depth between 2 and 6 mm, which was in the near field, had less artifacts and could be observed more clearly from the MNULA than from the MULA. The target points in the MNULA part of Figure 15b interfered with each other less, which provided a clearer imaging result with a small size of vision. Especially for target point *a*, the MULA caused the point integrate with other artifacts, while the MNULA eliminated the extra artifacts on both sides, making the target point more specific.

In this situation, it was clear that the less artifacts caused by the transducer, the smaller the sum of values of the reflected data around the target point. In order to better evaluate the ability to eliminate artifacts, the contrast difference could be estimated by introducing a point spread function (PSF) [8], which means the energy concentric with *R*. The energy for target point could be calculated from the definition of PSF. We defined the contrast equation as below:

(11) $$CTR_{point}=\frac{E\left(x_{point},y_{point},r_{0}\right)}{E\left(x_{point},y_{point},r\right)}\;\;(r\;>\;r_{0}\;)$$

where $CTR_{point}$ reflects the energy proportion between the target region and its nearby region, which contains artifacts; E is the total energy of a r diameter circle centered at ($x_{point},y_{point}$), where the target point lies; and $r_{0}$ is typically chosen as 5λ [25] to describe the effective region of the detected target point. The same circle center can effectively reduce the error caused by a slight position difference between target points in two images. During each calculation for the two images, r kept the same value to cover the same area around the target point position.

**Figure 16:**
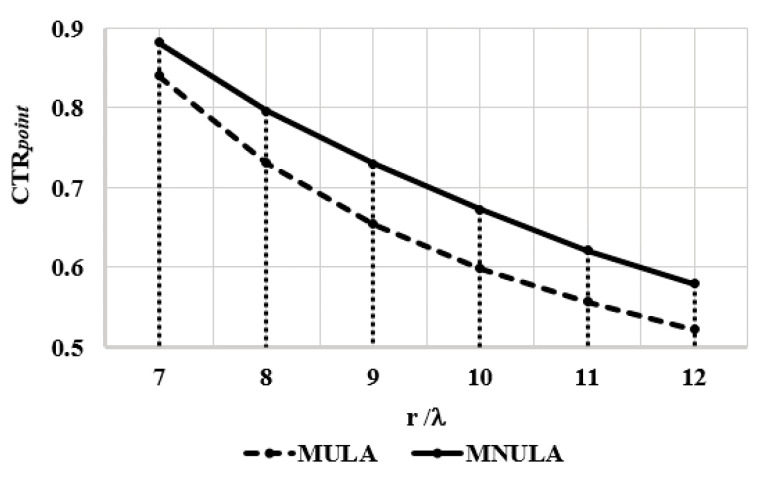
$CTR_{point}$ (the energy proportion between the target region and its nearby region) of different *r* values (the diameter of the circle of centered at ($x_{point},y_{point}$)). The dashed line is for the MULA, and the solid line is for the MNULA.

Take the target point *a* as an example. r was increased by λ ranging from *7*λ to *12*λ. The results are shown as Figure 16. As r increased, the trend in the diagram shows that the energy of the target point decreased and eventually reached a fixed value. The $CTR_{point}$ of the MULA was always smaller than that of the MNULA, which means there was less energy distributed in the selected area of the MNULA. Since the area of the selected was the same, the trend indicated that the amplitude of the artifacts was much lower in the MNULA.

**Figure 17:**
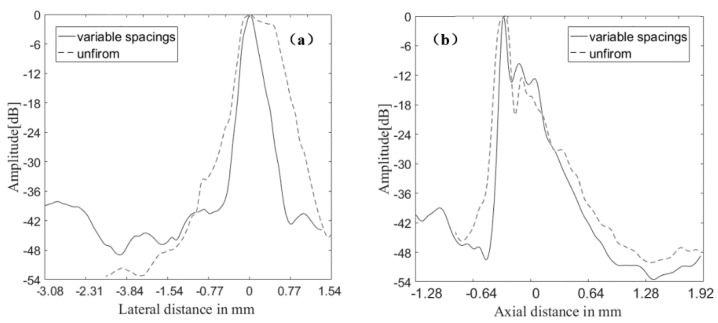
Lateral and axial envelope of echo signals of the point *a* acquired by (**a**) the MULA and (**b**) the MNULA.

The envelopes of the echo signals of the point *a* are presented in Figure 17. The axial and lateral resolutions were determined from the −6 dB envelope width as 144 and 603 μm for the MULA, respectively. For the MNULA, the axial and lateral resolutions were, respectively, 72 and 236 μm. It can be seen the imaging resolution was improved by changing the transducer structure.

All the experiment results and analysis have shown that the MNULA had the better ability to eliminate artifacts than the MULA, both in distribution and amplitude.

## 5. Discussion

In this work, we verified the improvements of the MNULA through a sound field and imaging simulation, as well as an imaging experiment on a real MNULA made by a laboratory. The reasons why the points detected by the MNULA were clearer and easier to observed than the MULA are as follows.

**Figure 18:**
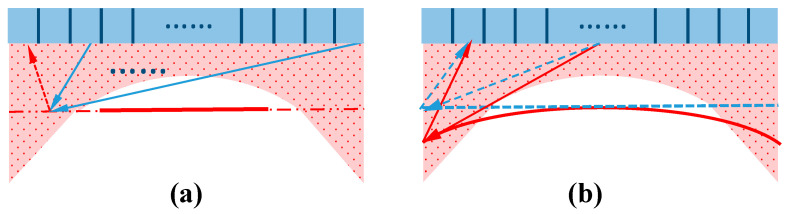
Two reasons why a uniform linear array causes error in CPWC: (**a**) discontinuous plane wave and (**b**) plane wave with a certain curvature. The blue line describes the ideal situation when all the elements of the existing MULA transmit and receive at the same time. The red line is the real situation. The red area indicates the possible imaging area with calculation error.

In the condition of a non-ideal plane wave, an error for CPWC is mainly caused by the shape of the plane wave. First of all, the shape of the plane wave in the near field emitted by the MULA is in fact discontinuous, which leads to the loss of echo information in some special positions. A theoretical diagram is shown as Figure 18a.

The process of beamforming selects and calculates the actual reflected data corresponding to the position of array elements. Thus, it is easy to predict that a lack of information reduces the accuracy of data screening. In the red region, the discontinuity of the isobaric surface is much more serious than that of other regions. In this area, the amount of information reflected back to the receiving elements is lesser than that in the other imaging region. That is why the reconstruction of the data points in this part produces some artifacts.

The details in simulation results of the sound field could also provide evidence for the above theoretical analysis. A simulation of the sound field of the MNULA showed that at a distance of 4 mm from the transducer, the energy distribution was uniform and regular. When the energy level was as low as $1\times 10^{4}$ Pa, the waveform was close to the shape of the theoretical plane wave. Thus, the image quality in the near field was improved, but the artifacts could not be completely eliminated.

The second reason that the points detected by the MNULA were clearer and easier to observed than the MULA was the curvature of plane wave. It was mentioned in Section 2 that the curvature was infinitely close to zero. However, the plane wave emitted by the MULA was actually not planar enough. It had a certain radian that could not be neglected. In this case, there were some differences between the actual propagation path and the ideal plane wave. The process is illustrated in Figure 18b. For an ideal case, a plane wave should be simultaneously emitted by each array element and form a shape like the blue dashed line in Figure 18b. If there are scattering points in the red part, the propagation trajectory and time delay calculation are supposed to satisfy the blue dashed arrows. Meanwhile, the actual wave at the edges forms slower than that in the middle. Thus, it takes more time for the actual waveform expressed in red arrows to travel to the points in the red region. The time error is too incalculable to be removed by CPWC.

The results of the sound field simulation in Section 3 showed that the shape of wave fronts was smoother and flatter. The waveform gradually stabilized and covered a wider field of view. Therefore, in the region above 10 mm, the artifacts on both sides disappeared due to the widening of the sound field. However, it should be noticed that the propagation also caused the energy to gradually diverge, and this may have decreased the resolution of imaging in this area.

In the experiment, the size of the handmade arrays was rather small, with an overall length of about 4 mm; this means the radius of a designed cyst should be less than 2 mm, which is a size that is currently rather difficult to apply to a cyst phantom. Thus, for the micro 16-element handmade array, we chose a dot imaging experiment rather than a cyst imaging experiment.

In this research, we only verified some simple structures of MNULAs. Theoretically, there are still more complex structural designs for MNULAs, and they can have better sound fields [26,27,28]. However, due to the limitations of time and cost, an optimal solution was been presented in this paper. Different structural arrangements for better signal transmission and receiving effects will be further studied in the future.

Additionally, there is still a long way to go before imaging quality becomes satisfactory. We made some improvements on the probe used in the traditional ultrasonic plane wave imaging process and improved the traditional plane wave algorithm according to the improvements of the probe, which was a major focus of the study. As far as we know, there has not been any significant breakthrough in the receiving and transmitting sequences of plane wave ultrasound imaging. The receive aperture control in near-field imaging and some other methods for receiving and transmitting sequences of plane wave ultrasound imaging can also be further studied in the future. All the results that we achieved could become the basis for further research on plane wave endoscopic imaging, B-mode imaging, or even the whole field of ultrasound transducers.

## 6. Conclusions

In this paper, we proposed a new structure type called an MNULA for ultrasound plane wave imaging, and we studied the properties of the MNULA in detail. The MNULA makes a structural improvement on the existing MULA. Through a simulation of a sound field and imaging, the advantages in sound field distribution and imaging quality were theoretically verified. This process helped get suitable parameters for an experiment. The prototype transducer was manufactured by our laboratory, and a phantom experiment demonstrated the improvements of the MNULA over the MULA in practical imaging. We then analyzed the differences between plane wave imaging theory and the actual sound field that the ultrasound linear array transmitted, thus explaining why the MNULA was able to bring improvements in image quality.

## Figures and Tables

**Table 1:** The structural parameters of simulation transducer models.

Structural Parameters	Uniform	Nonuniform1	Nonuniform2	Nonunifrom3
Kerf1 (μm)	25	25	25	25
No. of kerf1	8	8	4	4
Kerf2 (μm)	—	—	15	15
No. of kerf2	—	—	4	4
Width1 (μm)	300	300	300	300
No. of width1	8	4	8	4
Width2 (μm)	—	200	—	200
No. of width2	—	4	—	4

**Table 2:** The structural parameters of the laboratory-made transducer.

Structural Parameters	MULA	MNULA
Kerf1 (μm)	25	15
No. of kerf1	8	8
Kerf2 (μm)	25	25
No. of kerf2	8	8
Width1 (μm)	300	300
No. of width1	8	8
Width2 (μm)	300	300
No. of width2	8	8

## Data Availability

No new data were created or analyzed in this study. Data sharing is not applicable to this article.
